# Supplementary material for: A drug-inducible sex-separation technique for insects
Source: Nat Commun. 2020 Apr 30;11:2106. doi: 10.1038/s41467-020-16020-2 (PMC7193620; doi:10.1038/s41467-020-16020-2)
Supplement: Supplementary file 1 — Supplementary Information [file 41467_2020_16020_MOESM1_ESM.pdf]

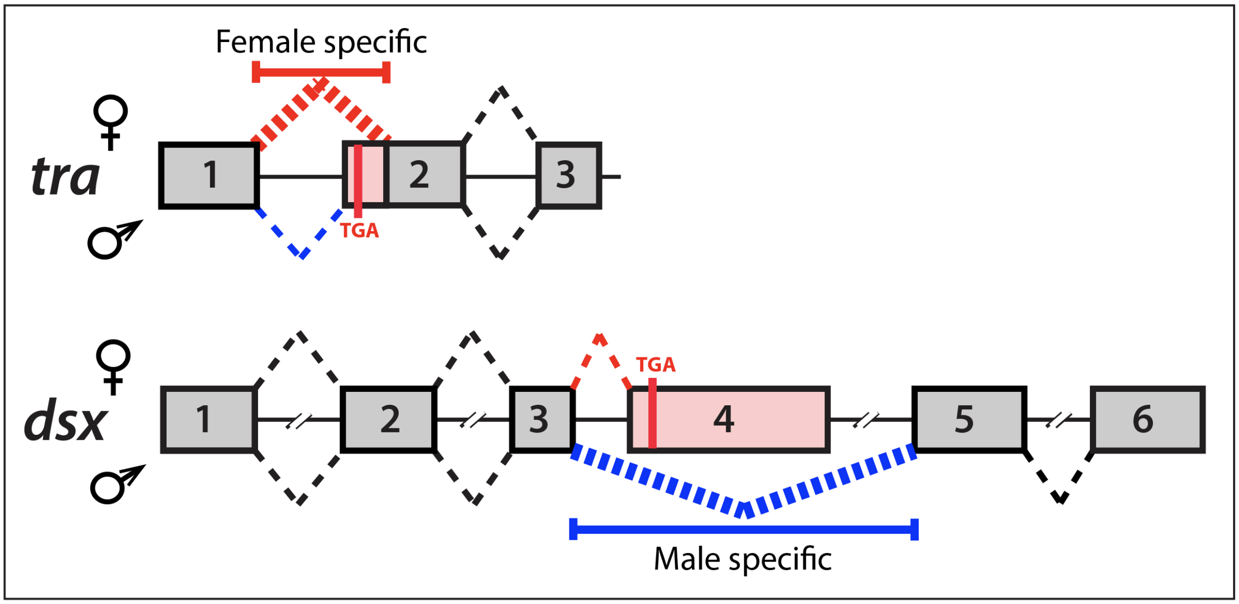

Supplementary Fig 1 Sex-specific alternative splicing of *Drosophila transformer* (*tra*) and *double sex* (*dsx*) genes. The female-specific intron between *tra* exons 1 and 2 (*traF*) is spliced out entirely in females, while in males some sequence remains, producing a stop codon and resulting in a premature termination of the TRA protein[44]. Inversely, the male-specific intron between *dsx* exons 3 and 5 (*dsxM*) is spliced out in males, while in females only its small part is spliced out and an entire exon 4 carrying a premature stop codon remains[45]. To establish female- or male-specific expression of antibiotic-resistance genes, we inserted a *traF* or *dsxM* intron sequence into the coding sequence of *PuroR* and *NeoR*.
