# Supplementary material for: A drug-inducible sex-separation technique for insects
Source: Nat Commun. 2020 Apr 30;11:2106. doi: 10.1038/s41467-020-16020-2 (PMC7193620; doi:10.1038/s41467-020-16020-2)
Supplement: Supplementary file 6 — Reporting Summary [file 41467_2020_16020_MOESM6_ESM.pdf]

nature research

Corresponding author(s): NCOMMS-19-41319A

Last updated by author(s): Apr 3, 2020

Reporting Summary

Nature Research wishes to improve the reproducibility of the work that we publish. This form provides structure for consistency and transparency in reporting. For further information on Nature Research policies, see Authors & Referees and the Editorial Policy Checklist .

Statistics

For all statistical analyses, confirm that the following items are present in the figure legend, table legend, main text, or Methods section.

n/a	Confirmed	
☐	☒	The exact sample size (*n*) for each experimental group/condition, given as a discrete number and unit of measurement
☐	☒	A statement on whether measurements were taken from distinct samples or whether the same sample was measured repeatedly
☐	☒	The statistical test(s) used AND whether they are one- or two-sided<br>*Only common tests should be described solely by name; describe more complex techniques in the Methods section.*
☒	☐	A description of all covariates tested
☐	☒	A description of any assumptions or corrections, such as tests of normality and adjustment for multiple comparisons
☐	☒	A full description of the statistical parameters including central tendency (e.g. means) or other basic estimates (e.g. regression coefficient) AND variation (e.g. standard deviation) or associated estimates of uncertainty (e.g. confidence intervals)
☐	☒	For null hypothesis testing, the test statistic (e.g. *F*, *t*, *r*) with confidence intervals, effect sizes, degrees of freedom and *P* value noted<br>*Give P values as exact values whenever suitable.*
☒	☐	For Bayesian analysis, information on the choice of priors and Markov chain Monte Carlo settings
☒	☐	For hierarchical and complex designs, identification of the appropriate level for tests and full reporting of outcomes
☒	☐	Estimates of effect sizes (e.g. Cohen's *d*, Pearson's *r*), indicating how they were calculated

Our web collection on statistics for biologists contains articles on many of the points above.

Software and code

Policy information about availability of computer code

Data collection	N/A
Data analysis	Statistical analysis was performed in JMP 8.0.2 by SAS Institute Inc.

For manuscripts utilizing custom algorithms or software that are central to the research but not yet described in published literature, software must be made available to editors/reviewers. We strongly encourage code deposition in a community repository (e.g. GitHub). See the Nature Research guidelines for submitting code & software for further information.

Data

Policy information about availability of data

All manuscripts must include a data availability statement. This statement should provide the following information, where applicable:

- Accession codes, unique identifiers, or web links for publicly available datasets
- A list of figures that have associated raw data
- A description of any restrictions on data availability

All data that are represented fully within the tables and figures. The plasmids constructed in the study (Figure 1c) were deposited at Addgene.org (#131613 – #131618). The homozygous sex-sorter lines was deposited at Bloomington Drosophila Stock Center (#79015). The remaining Drosophila lines will be made available upon request.

Field-specific reporting

Please select the one below that is the best fit for your research. If you are not sure, read the appropriate sections before making your selection.

Life sciences study design

All studies must disclose on these points even when the disclosure is negative.

Sample size	We counted all flies emerged from the eggs laid by a replicate group of parents flies on the food with and without drug supplementation.
Data exclusions	All data were included.
Replication	We used different parent fly groups randomly picked up at different times during a six-month period for each replicate from the same transgenic (random insertion site) line or control flies to lay eggs into the food with or without drug. At least three (but in most cases, five) biological replicates that originated from separate generations of each line were used to estimate means and ± one SD.
Randomization	Random groups of around 20 flies picked up over a six-month period from transgenic and control lines (over several generations) were used for tests, to lay eggs on the food.
Blinding	No. It was not necessary since we used different group flies (at the different times / generations) from each transgenic line and we did this for 2 or different insertion lines.

Reporting for specific materials, systems and methods

We require information from authors about some types of materials, experimental systems and methods used in many studies. Here, indicate whether each material, system or method listed is relevant to your study. If you are not sure if a list item applies to your research, read the appropriate section before selecting a response.

Materials & experimental systems

n/a	Involved in the study	
☒	☐	Antibodies
☒	☐	Eukaryotic cell lines
☒	☐	Palaeontology
☐	☒	Animals and other organisms
☒	☐	Human research participants
☒	☐	Clinical data

Methods

n/a	Involved in the study	
☒	☐	ChIP-seq
☒	☐	Flow cytometry
☒	☐	MRI-based neuroimaging

Animals and other organisms

Policy information about studies involving animals; ARRIVE guidelines recommended for reporting animal research

Laboratory animals	Drosophila melanogaster w[1118] as a wildtype control and random transgenic lines generated from the control ine.
Wild animals	N/A
Field-collected samples	N/A
Ethics oversight	We have complied with all relevant ethical regulations for animal testing and research, and conformed to the UCSD institutionally approved biological use authorization protocol (BUA #R2401).

Note that full information on the approval of the study protocol must also be provided in the manuscript.
