# Supplementary material for: A drug-inducible sex-separation technique for insects
Source: Nat Commun. 2020 Apr 30;11:2106. doi: 10.1038/s41467-020-16020-2 (PMC7193620; doi:10.1038/s41467-020-16020-2)
Supplement: Supplementary file 7 — Description of Supplementary Data Files [file 41467_2020_16020_MOESM7_ESM.pdf]

**Title: Supplementary Data 1:** Development of sex-sorter system.
**Description:** This supplementary excel file contains the raw count data underlying Fig. 1b–c and Fig. 2b–c.

**Title: Supplementary Data 2:** Titration of drug concentration for the homozygous sex-sorter line.
**Description:** This supplementary excel file contains the raw count data underlying Fig. 3a–b.

**Title: Supplementary Data 3:** Survival rate of the homozygous sex-sorter line.Description:
**Description:** This supplementary excel file contains the raw count data / analysis underlying Fig. 3c–d.
